# Does the periportal end of a double-lumen endobronchial tube need to be fixed to prevent dislocation of the cuffed end caused by a change in position? A randomized controlled trial

**DOI:** 10.1080/07853890.2023.2247422

**Published:** 2023-08-24

**Authors:** Zhi-yuan Chen, Yu-mei Lin, Jian-hua Wu, Yu-yu Fu, Xiao-ting Xu, Yan Li, Li-hong Chen, Li-ming Xu

**Affiliations:** Department of Anesthesiology, The Second Affiliated Hospital of Fujian Medical University, Quanzhou, China

**Keywords:** Intubation, dislocation, poor alignment, double-lumen endobronchial tube, one-lung ventilation

## Abstract

**Objective:**

This study aimed to evaluate the effects on the dislocation and misalignment of the cuffed end of a double-lumen endobronchial tube (DLT) when a patient moves from a horizontal to a lateral position without fixation.

**Methods:**

A total of 148 patients who had undergone video-assisted thoracoscope surgery were enrolled and randomly divided into two groups: a group in which the periportal end of the DLT was fixed with tape (group I; *n* = 74) and a group in which the periportal end of the DLT remained unfixed (group II; *n* = 74). Both groups were given an intravenous induction for double-lumen endobronchial intubation and then moved from a horizontal position to a lateral position, after which the alignment of the bronchial cuffed end of the DLT was assessed using a fiberoptic bronchoscope.

**Results:**

After lateral position, the dislocation rate of group I and group II was 44.6% and 20.2%, and the misalignment rate was 27.0% and 8.1%, respectively, the incidence of dislocation and misalignment was significantly lower in group II than in group I after the change to a lateral position (*p* < 0.05). After lateral position, the total rate of airway injury was 25.7% in group I and 5.4% in group II, the incidence of airway injury was significantly lower in group II than in group I (*p* < 0.05), as was the incidence of sore throat, hoarseness, and cough on postoperative day 1 (*p* < 0.05). The average outward dislocation of the periportal end of the DLT in group II was 1.5 cm.

**Conclusion:**

A DLT without periportal fixation is less likely to be displaced and poorly aligned when the patient moves from a horizontal to a lateral position, which could facilitate intra-operative management and reduce the incidence of postoperative complications.

## Key messages

Through a randomized controlled trial, this study innovatively found that no double-lumen endobronchial tube (DLT) peripheral tape binding can prevent the dislocation and misalignment of the DLT bronchial cuffed end in patients undergoing thoracic surgery from horizontal to lateral position.

## 1. Introduction

A double-lumen endobronchial tube (DLT) is usually used to perform lung isolation and one-lung ventilation (OLV) in patients undergoing thoracic surgery. It can help with the accurate isolation of both lungs and has the advantages of good surgical field exposure and effective suctioning of secretions and blood [1], thereby creating good conditions for thoracic surgery. However, clinical application is based on the accurate alignment of the bronchial cuffed end of the DLT with the bronchial opening. The widely used Robertshaw DLT without a carinal hook is relatively simple to operate and less damaging than other types of DLT because the front end of the tube enters the glottis during tracheal intubation without the direction of the tube needing to be changed [2,3]. However, video-assisted thoracoscope surgery often needs to be performed with the patient in a lateral position, and the process of moving the patient from a horizontal to a lateral position is likely to cause the dislocation of the bronchial cuffed end of the DLT, resulting in misalignment with the bronchial opening. This not only leads to the failure of OLV, causing increased airway ventilation pressure, intraoperative hypoxia, and impaired carbon dioxide expulsion, but also increases the incidence of postoperative airway and pulmonary complications [4,5]. Many researchers have studied how to reduce the dislocation of the bronchial cuffed end of the DLT and its poor alignment with the bronchial opening during a position change, and some results have been achieved [6,7]. The suggested methods almost always address the problem by limiting the position changes of the head and neck, but a change in the position of the patient’s head and neck during a shift from a horizontal to a lateral position is inevitable. Therefore, the present study aimed to identify an easier and more effective way to prevent the dislocation and misalignment of the DLT by observing the effect of using a DLT without periportal fixation.

## 2. Materials and methods

### 2.1. General characteristics

A total of 148 cases that met the American Society of Anesthesiologists (ASA) classification I–III and who had been selected for VATS in our hospital between December 2019 and November 2020 were enrolled. Of these, 78 cases required left-sided intubation and 70 required right-sided intubation. The sample included 82 males and 66 females aged between 28 and 76 and with a body mass index (BMI) of 18–25 kg/m2, mean (22.1 ± 3.5) kg/m2. Patients with thoracic deformities, tracheal abnormalities, or main airway stenosis, tumors, and tracheoesophageal fistulas were excluded.

Preoperative informed consent was given by all patients and their relatives, and approval was obtained from the Ethics Committee of the Second Hospital of Fujian Medical University (2019 Fujian Medical University Second Ethical Review no. 215). The study was registered in the Chinese Clinical Trial Registry with registration no. ChiCTR1900027200. Reporting of the study conforms to broad EQUATOR guidelines [8].

### 2.2. Grouping

Statistical Analysis System (SAS, USA) software was used to generate the random sequence numbers for grouping, and the patients were divided into groups I or II at a ratio of 1:1, with 74 cases in each group. The grouping information was sealed in envelopes and randomly distributed to the subjects. The envelopes were opened by nurses on the day of surgery to check the random serial numbers and inform the anesthesiologists, all of whom had more than 10 years of clinical experience. The patients in group I received adhesive tape fixation at the periportal end of the DLT, while those in group II remained unfixed.

### 2.3. Methods of anesthesia

#### 2.3.1. Induction of anesthesia

All patients were given an intravenous infusion of 0.01 mg/kg of penehyclidine hydrochloride 10 min before being anesthetized, and anesthesia was induced intravenously with 2.0 mg/kg of propofol, 0.3–0.5 μg/kg of sufentanil, and 0.2 mg/kg of cisatracurium. When the bispectral index (BIS) value was close to 50, the DLT was directly inserted with the help of a fiberoptic bronchoscope, and its position was determined. The DLT was then fixed after verification of good alignment between the bronchial cuffed end of the DLT and the bronchial opening, with a tracheal cuff inflation pressure of 40 mmHg for large cuffs and 35 mmHg for bronchial cuffs. The depth of the DLT position and the alignment of the opening were recorded.

**Figure 1.**
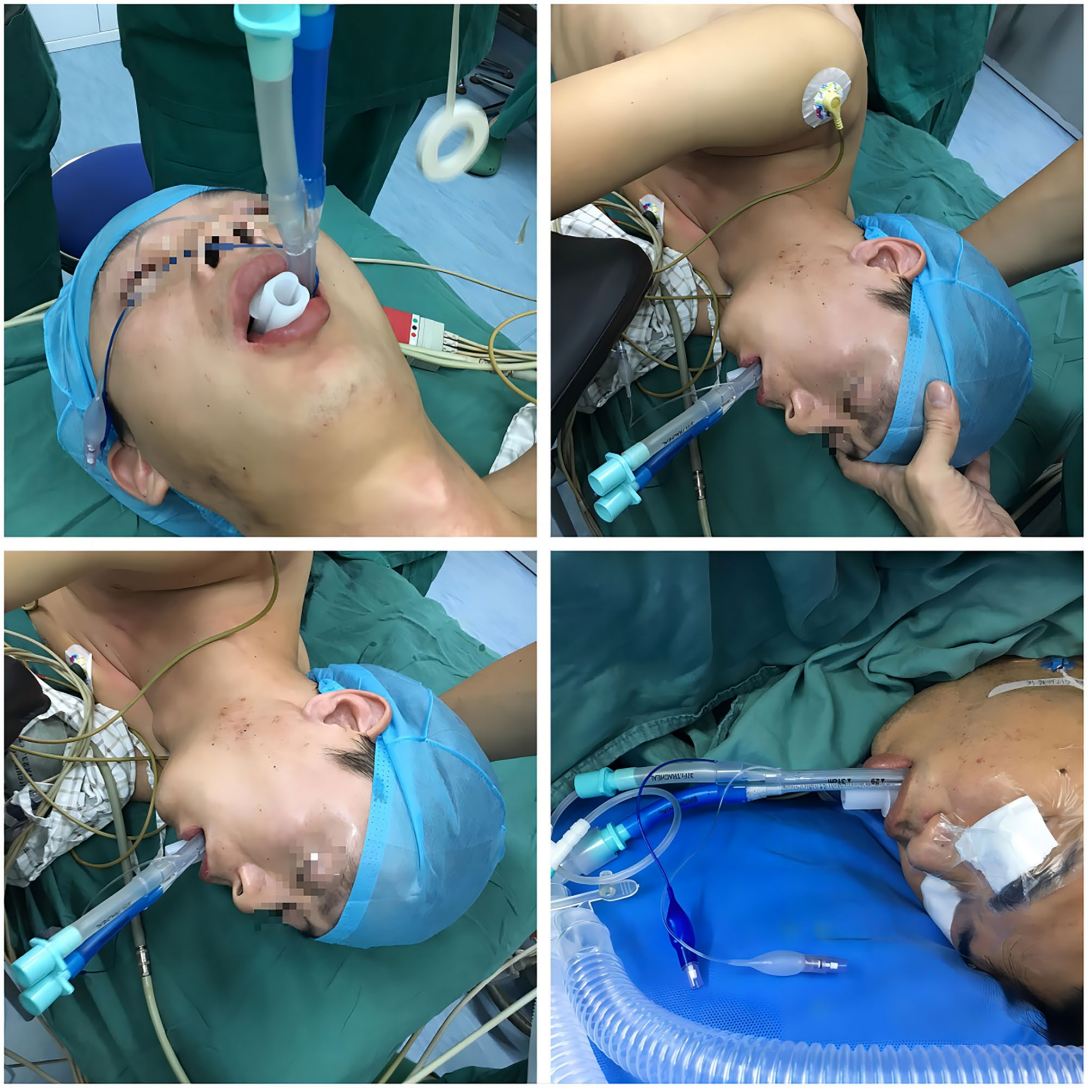
Group II patients without perioral duct tape fixation.

**Figure 2.**
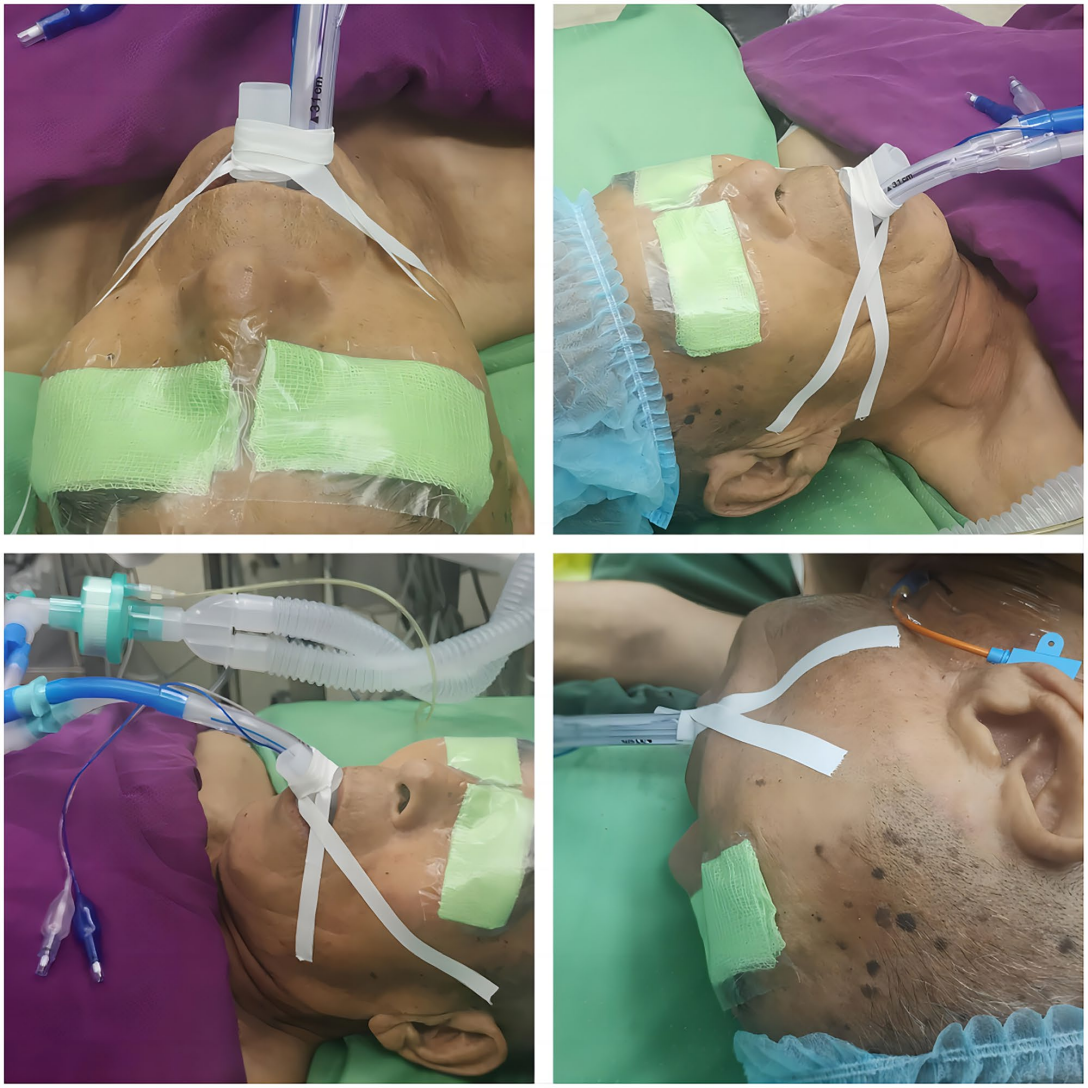
Group I patients with perioral duct tape fixation.

In group I, dental pads were placed in the traditional way and secured with adhesive tape at the periportal end of the DLT. Specifically, after the end of the DLT was accurately positioned by fiberoptic bronchoscopy, a plastic dental pad was placed between the maxillary and mandibular teeth of the patient ‘s mouth, and then two cloth tapes with a width of 1 cm and a length of 35 cm were used to bind the DLT and the plastic dental pad for two laps, and then the ends of the tape were attached to the skin of the patient ‘s cheek. All patients in group I were operated by the same anesthesiologist. While in group II, dental pads were inserted without periportal fixation of the DLT, allowing the tube to slide freely and smoothly between the maxillary and mandibular teeth. In the process of patients changing from horizontal position to lateral position, whether it is group I or group II, the anesthesiologist cooperates with the surgeon to take both hands to hold the patient ‘s head for axial rotation of 90 degrees. In group II, a plastic dental pad is routinely placed in the oral cavity to open the upper and lower incisors, so that the DLT can slide freely between the maxillary and mandibular teeth, and the anesthesiologist does not need to specially fix the catheter to prevent displacement. Patients in both groups were then moved from a horizontal to a lateral position after 10 min, and a Tiro anesthesia machine (Drager, Germany) was connected for mechanical ventilation with pure oxygen. See Figures 1,2.

#### 2.3.2. Maintenance of anesthesia

A total of 1% sevoflurane was administered intraoperatively, with an inhalation oxygen flow rate of 2 L/min, and the patient’s BIS value was maintained at 45–55 by adjusting the effective chamber target concentration of propofol (± 0.5 μg/ml, up to 3.5 μg/ml). The target concentration of the effective chamber of remifentanil was adjusted to 1.5–4.0 ng/ml, and the fluctuation of the mean arterial pressure (MAP) was maintained at no more than ± 20% of the baseline value. When necessary, 5 μg of sufentanil was injected intravenously, and an intermittent intravenous injection of 5 mg of cisatracurium was administered to maintain muscle relaxation. The respiratory parameters were adjusted to maintain the partial pressure of end-tidal carbon dioxide at 35–40 mmHg. The inhalation of sevoflurane was stopped 10 min before the end of surgery, and the intravenous general anesthetic was terminated 5 min before the end of surgery.

### 2.4. Selection of the DLT

Based on the endotracheal diameter (taken at the level of the sternoclavicular joint), which was measured preoperatively by chest computed tomography, the DLT model was determined by selecting the one with the largest diameter possible for the patient. When the tracheal inner diameter measurement value was ≥ 17 mm, a 39 Fr was selected; when the tracheal inner diameter was ≥ 15 mm, a 37 Fr was selected; when the tracheal inner diameter was ≥ 13 mm, a 35 Fr was selected; and when the tracheal inner diameter was ≥ 11 mm, a 33 Fr was selected. The bronchial cuff was inflated by 2–4 ml and the tracheal cuff by 6–8 ml to prevent air leakage. If the inflation volume was greater or lower than the above range, the tube selection was considered inappropriate and excluded from the study.

### 2.5. Determination of DLT position and observation indicators

#### 2.5.1. Determination of DLT bronchial cuffed end dislocation

Before intubation, a mark (L 0.5) was made on the DLT at 0.5 cm from the upper edge of the bronchial capsule. The patient was moved from a horizontal to a lateral position 10 min after double-lumen endobronchial intubation, as required for the surgery, at which time fiberoptic bronchoscopy was performed again and the position of the DLT determined. The DLT’s position was adjusted so that the L 0.5 line was level with the carinal in the normal position. If the position deviation exceeded 0.5 cm, the DLT was judged to be dislocated at the bronchial cuffed end [8].

#### 2.5.2. Determination of DLT bronchial cuffed end misalignment

After the patient was moved from a horizontal to a lateral position, the position of the DLT was checked again using fiberoptic bronchoscopy. Misalignment in patients with a left-sided DLT was identified as failure to completely display the bronchial openings of the left upper and lower lung lobe through the left double-lumen bronchial opening. Misalignment in patients with a right-sided DLT was identified as failure to completely display the middle and lower lung lobe openings through the right double-lumen bronchial opening, as well as failure to display the right upper lung lobe bronchial opening in the lateral aperture. Bronchial cuffed end dislocation and misalignment were determined and recorded and then corrected in a timely manner. The DLT in the patients in group II was fixed with adhesive tape, and the tube cuff pressure was readjusted to 28 mmHg for the tracheal cuff and 25 mmHg for the bronchial cuff in both groups [8].

#### 2.5.3. Hemodynamic changes after moving to a lateral position

All patients underwent flexure artery puncture and tube placement and were connected to monitors to allow the observation of invasive arterial pressure in real time. The MAP and heart rate (HR) were observed and recorded before (T0) and after (T1) the patient was moved to a lateral position.

#### 2.5.4. Injury to the tracheal carina and bronchial mucosa

The patient was returned to a horizontal position at the end of the operation, and the DLT was removed after reaching the extubation standard. After extubation, fiberoptic bronchoscopy was performed again to check for injury to the tracheal carina and bronchial mucosa. Injury was graded as mild (a little mucous membrane with scattered congestion and red swelling), moderate (mucous membrane with obvious congestion, red swelling, or a little oozing blood), or severe (airway mucous membrane with obvious oozing blood or with active bleeding). All of the above involving the use of fiberoptic bronchoscopy to evaluate the position and displacement of DLT and the injury of carina and tracheal mucosa were performed by the same physician with rich experience in the use of fiberoptic bronchoscopy, and the physician was blind to the case grouping.

#### 2.5.5. Follow-up and recording of sore throat, hoarseness, and cough on the first day after surgery

A follow-up visit was conducted on the ward on the first day after surgery, and sore throat, hoarseness, and cough were recorded. Sore throat was graded [9] as mild (slight pain in the throat with a sensation of a foreign body), moderate (obvious pain when swallowing), or severe (persistent pain, with difficulty in swallowing). Hoarseness was graded as mild (low pitch), moderate (hoarse voice), or severe (loss of voice).

#### 2.5.6. Observation and recording of the dislocation of the periportal end of the DLT in group II

Adopting the flat maxillary incisor at the periportal end of the DLT as the zero point, the dislocation of the DLT relative to the incisor before and after the patient was moved to a lateral position was observed and recorded. The tube moving outward toward the incisor was recorded as a positive value, while the tube moving inward from the incisor was recorded as a negative value.

### 2.6. Sample size calculation and statistical methods

#### 2.6.1. Sample size calculation

The sample size was calculated according to the following formula:

$$n_{1}=n_{2}=1641.4\times {\left[\frac{\left(u_{\alpha }+u_{\beta }\right)}{\left(\sin^{-1}\sqrt{p_{1}}-\sin^{-1}\sqrt{p_{2}}\right)}\right]}^{2},$$

where n1 and n2 represented the sample sizes in group I and group II, respectively. The data reported by Klein et al. [10] were adopted as the rate of DLT dislocation in group I, which resulted in 93/200 = 46.5%. Based on the results of the pre-experiment, it was expected that the tube dislocation rate in group II could be reduced by at least 25%, and thus the tube dislocation rate in group II was taken as approximately 21.5%. Accordingly, assuming α = 0.05 and 1-β = 0.9, and using a two-sided test, the number of cases per group sample was calculated by SPSS 11.0 software to be at least 74 cases, so it was determined that the total number of cases for this study should be 148, with 74 cases in each group.

#### 2.6.2. Statistical analysis

The data were unblinded after the experiment and grouped for data processing. SPSS 11.0 software was used for statistical analysis. The measurement data were expressed as mean ± standard deviation ($\bar{\text{X}}$± S), and a grouped t-test was used for comparison between the groups. A χ^2^ test (adjusted χ^2^ test and Fisher’s exact probability method) was used for the comparison of countable data. *p* < 0.05 was considered statistically significant.

### 2.7. Study design

**Figure 3.**
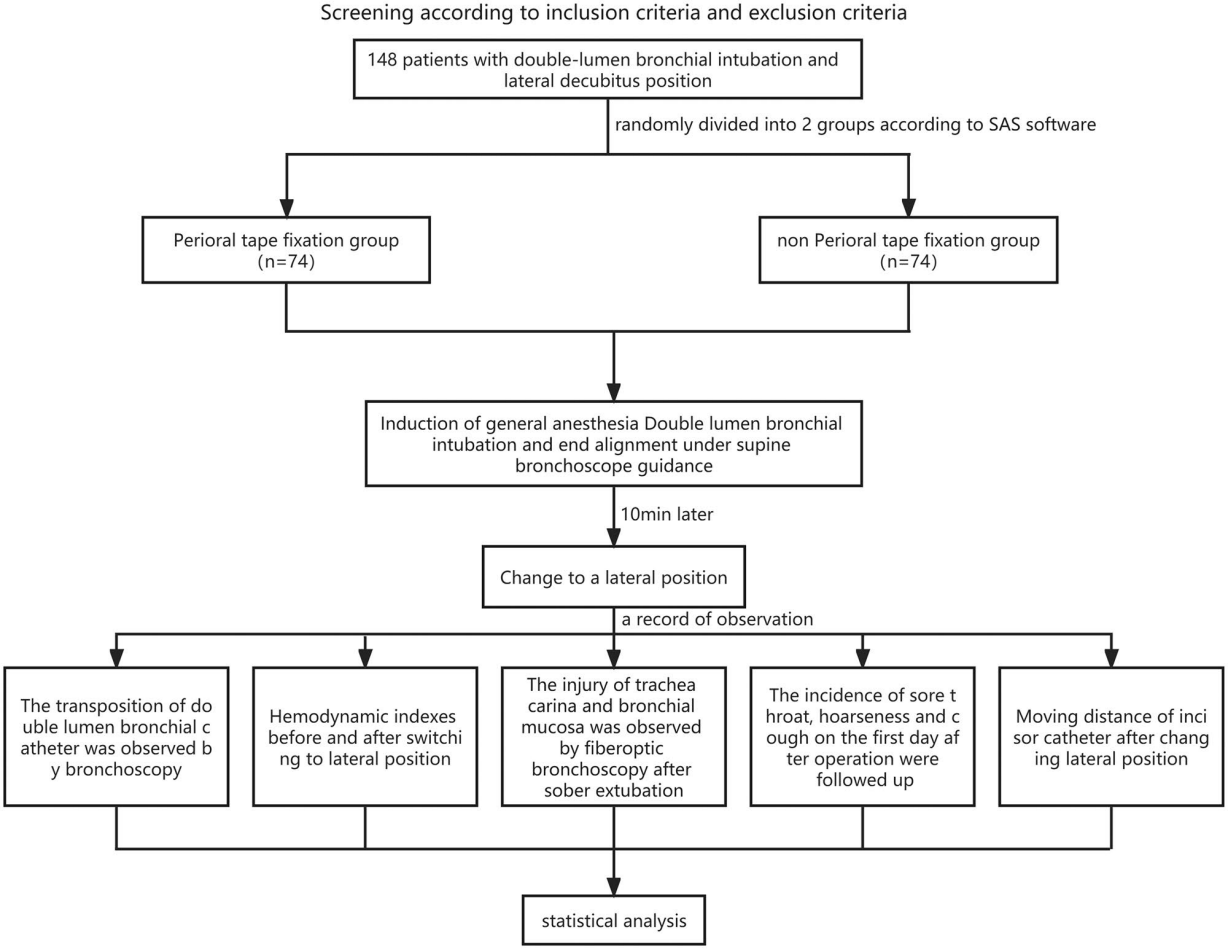
Study design drawing.

A total of 148 patients who needed to insert a DLT and changed their position were selected and randomly divided into the perioral adhesive tape fixation group and the non-fixation group. The dislocation and misalignment of the bronchial cuffed end of the DLT were observed after the position of the two groups changed from horizontal to lateral position, so as to evaluate the preventive effect of non-perioral adhesive tape fixation on the dislocation of the bronchial cuffed end of the DLT. See Figure 3.

## 3. Results

### 3.1. General characteristics

There were no statistically significant differences between the two groups in gender, age, height, BMI, endotracheal diameter, DLT model, ASA classification, and type of tube (*p* > 0.05), as shown in Table 1.

**Table 1.** The general characteristics of patients and comparison between the groups.

Group	number of cases	Gender (M/F)	Age (Years)	Height (cm)	Body mass (KG)	Endotracheal diameter (mm)	Type of tube (Fr)	Tube model Left/Right
Group I	74	42/32	53 ± 18.9	164.7 ± 11.2	59.±10.2	15 ± 1.7	37.1 ± 0.8	40/34
GroupII	74	40/34	49 ± 20.6	165.6 ± 10.9	60.±10.5	15 ± 1.6	36.8 ± 0.9	38/36

Note: *p* > 0.05 for the comparison between the two groups.

### 3.2. Comparison of dislocation and misalignment of the bronchial cuffed end of the DLT

**Figure 4.**
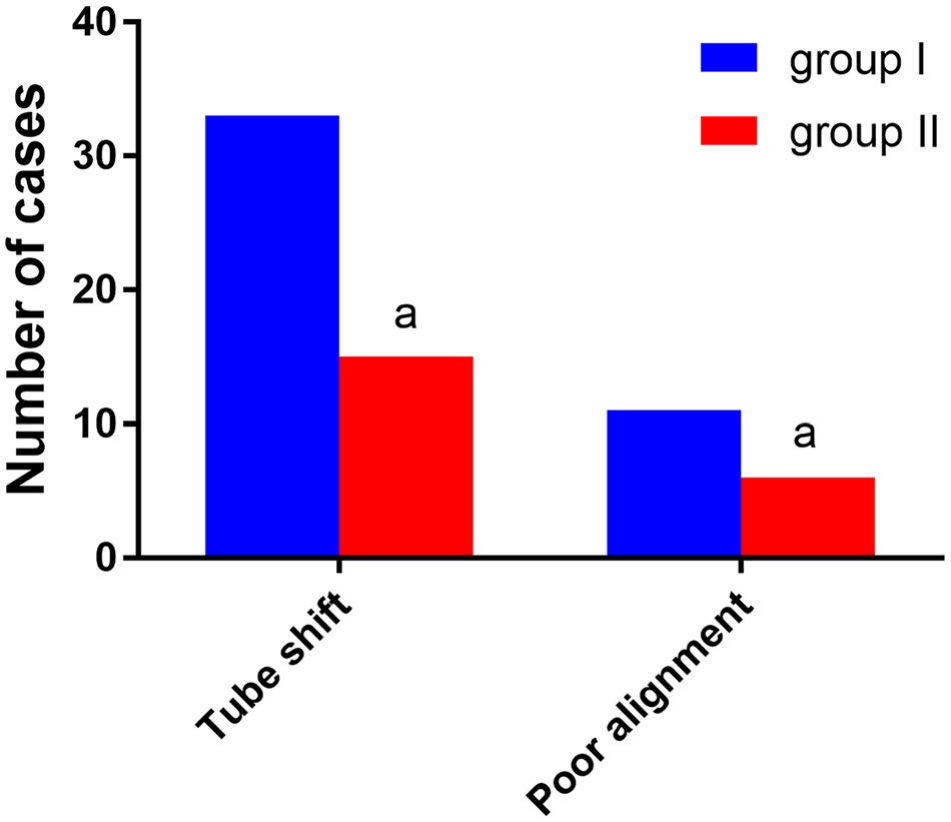
The cuff end dislocation and misalignment of the DLT after lateral Decubitus position in the two groups (*n* = 74). Note: Compared with group I, ^α^*P* < 0.05.

A total of 33 cases in group I and 15 cases in group II experienced dislocation of the cuffed end of the DLT when the patient’s position was changed from horizontal to lateral, and the difference between the two groups was statistically significant (*p* < 0.05). Furthermore, 11 cases in group I and six cases in group II experienced misalignment of the cuffed end of the DLT, and the difference between the two groups was statistically significant (*p* < 0.05). See Figure 4.

### 3.3. Comparison of MAP and HR

**Figure 5.**
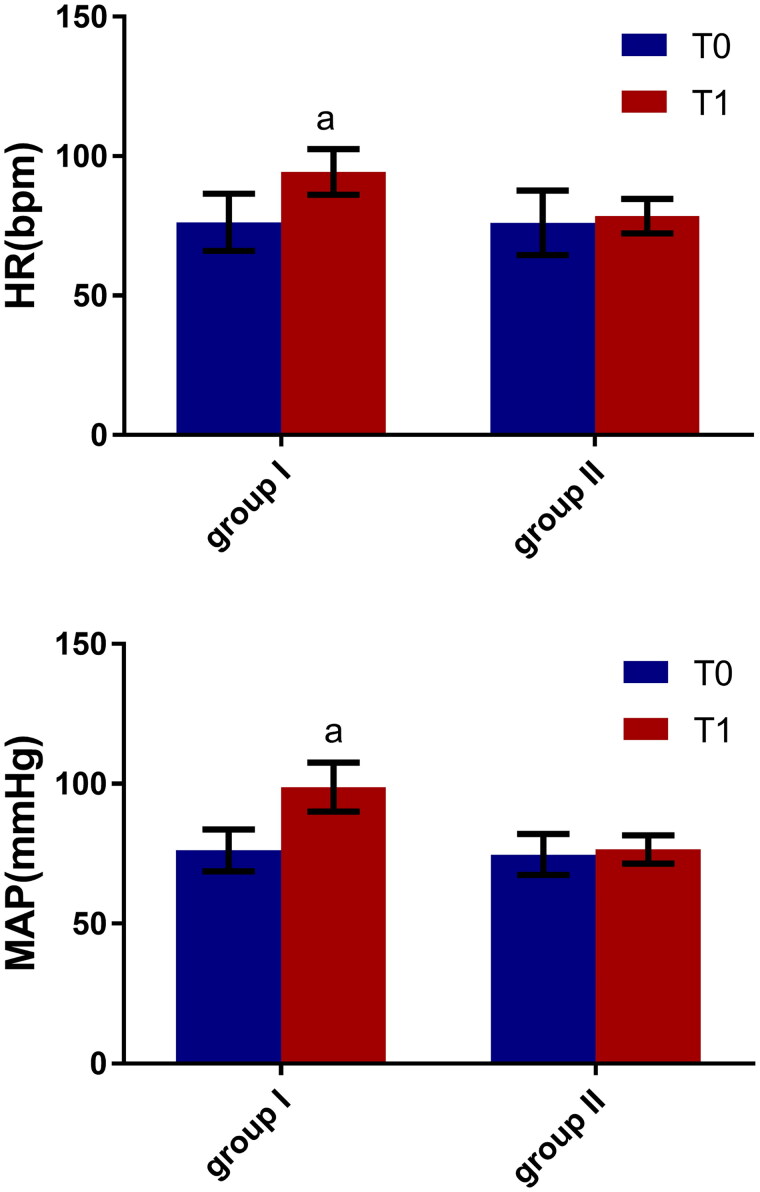
Hemodynamic changes before and after changing to a lateral position in both groups ($\bar{\text{X}}$± s), *n* = 74. Note: Compared with T0, ^α^*P* < 0.05.

It was found that MAP and HR were higher in both groups when the patients were moved from a horizontal to a lateral position. In group I, the differences in MAP and HR before and after the change in position were statistically significant (*p* < 0.05), but in group II these differences were not statistically significant (*p* > 0.05). See Figure 5.

### 3.4. Comparison of injury to the tracheal carina and bronchial mucosa

**Figure 6.**
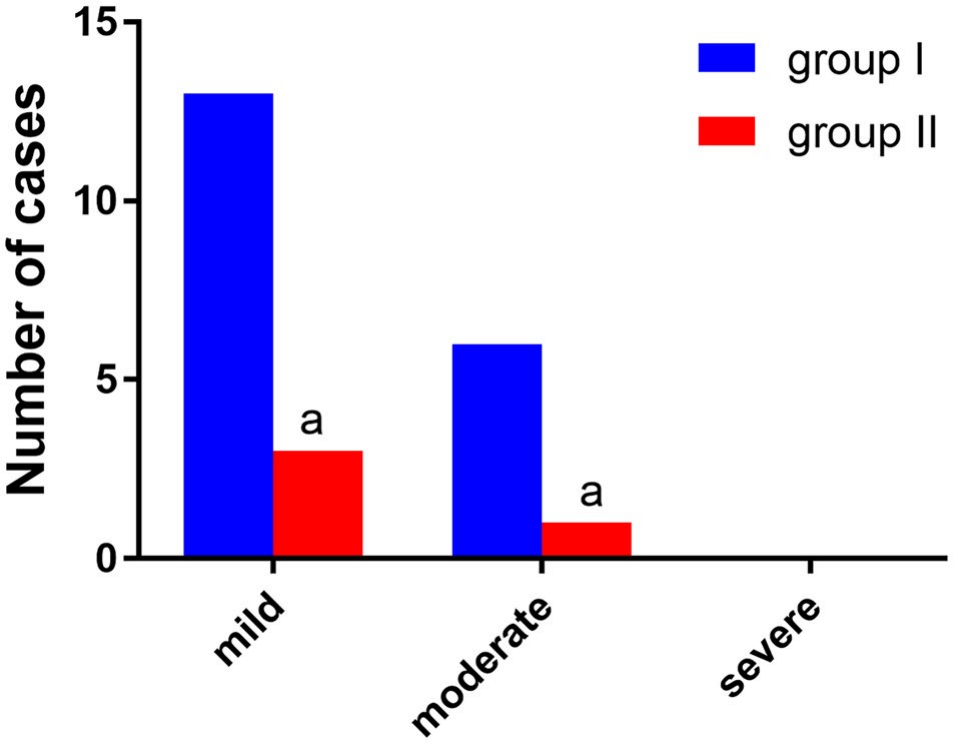
Comparison of injury to the tracheal carina and bronchial mucosa (*n*, %), *n* = 74. Note: Compared with group, ^α^*P* < 0.05.

**Figure 7.**
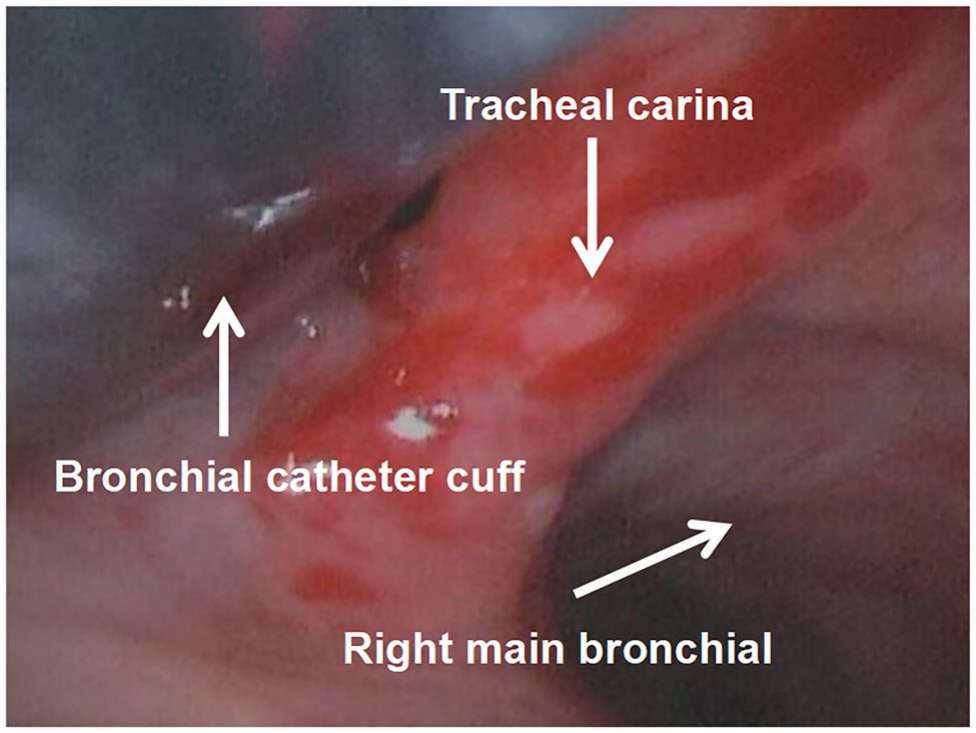
This is a Photograph of the tracheal mucosa of the patient in group I. it can be seen that the cuff end of the DLT is displaced outward, and the carinal mucosa is damaged, bleeding, and edema due to repeated catheter displacement.

**Figure 8.**
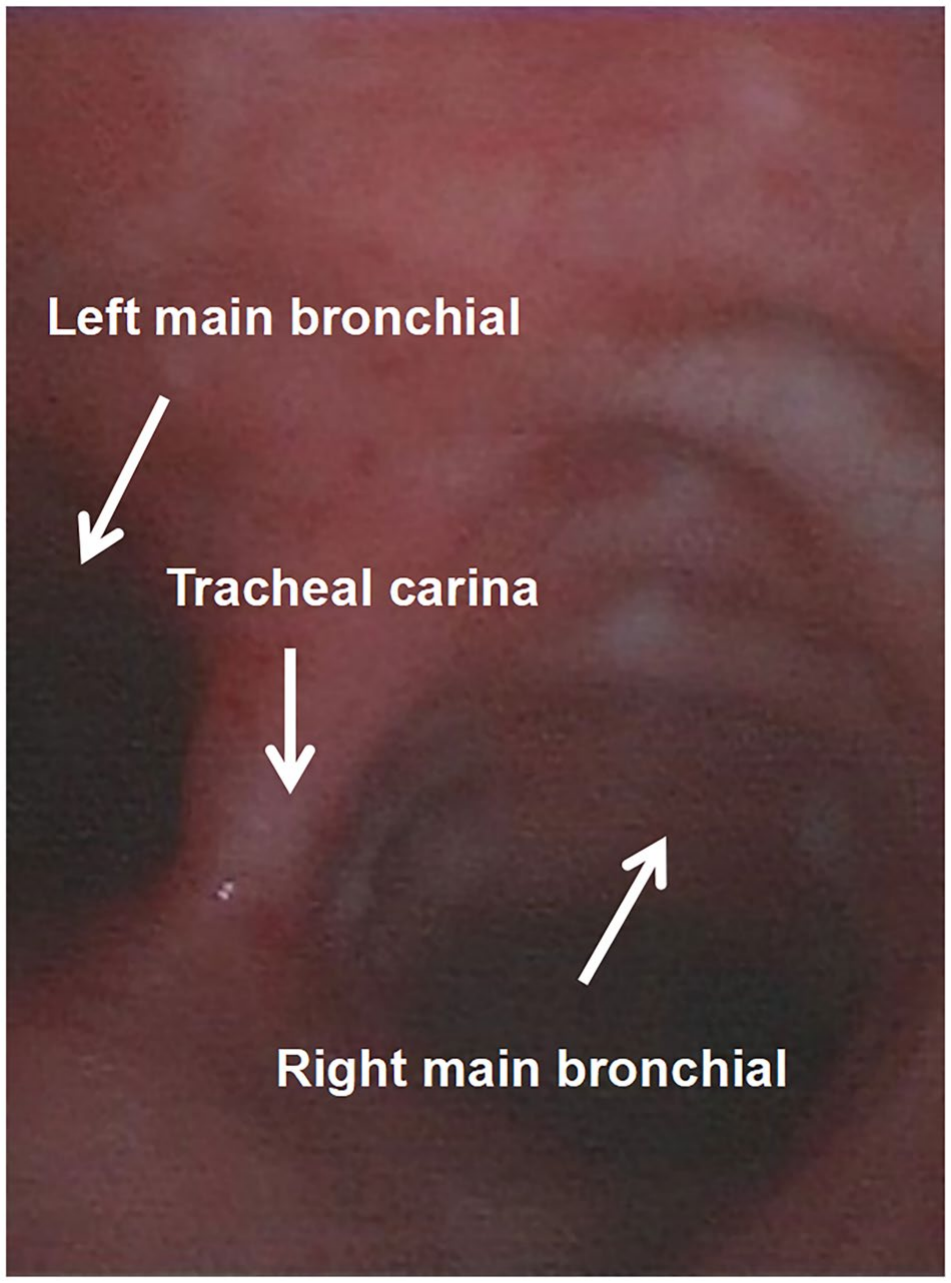
This is a Picture of the tracheal mucosa in group II, and it can be seen that the carinal and main bronchial mucosa have very little hemorrhage, damage, or edema.

Three cases of mild injury to the tracheal carina and bronchial mucosa were found in group II and 13 cases in group I, and there was one case of moderate injury in group II and six cases in group I. The difference between the two groups in mild injury and moderate injury was statistically significant (*p* < 0.05). There were no cases of severe injury in either group. See Figures 6–8.

### 3.5. Comparison of postoperative sore throat, hoarseness, and cough

**Figure 9.**
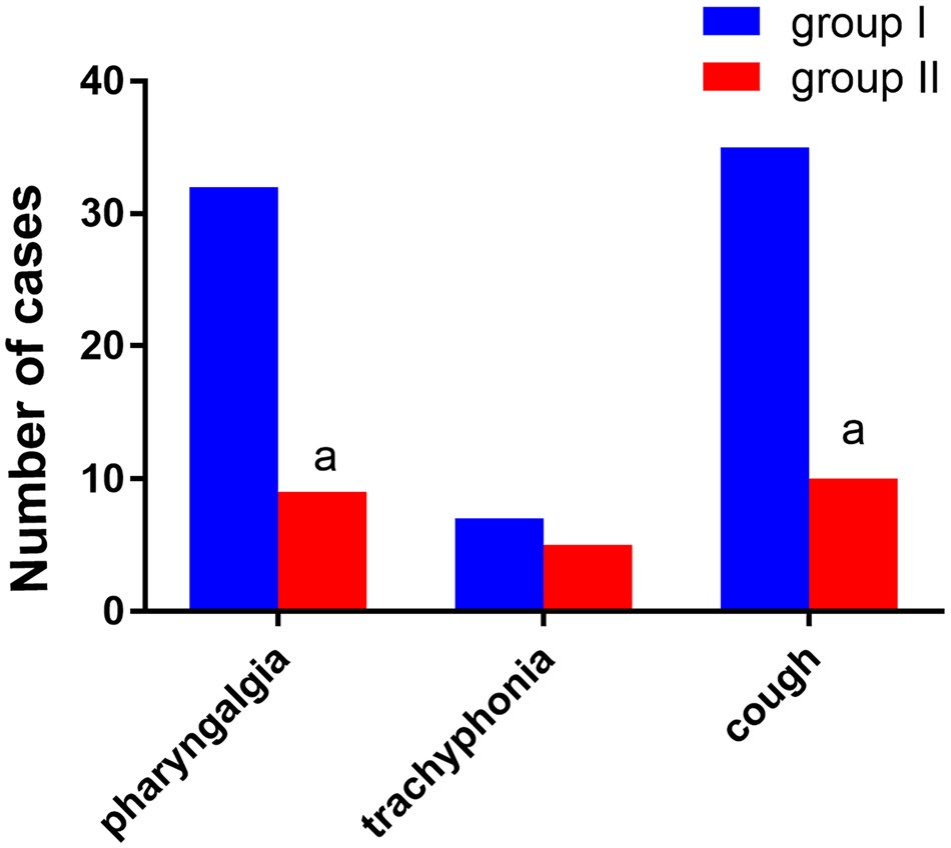
Comparison of postoperative sore throat, hoarseness, and cough between the two groups of patients (*n*, %), *n* = 74. Note: Compared with group I, ^α^*P* < 0.05.

There were nine cases of sore throat in group II and 32 cases in group I, and the difference between the two groups was statistically significant (*p* < 0.05). There were seven cases of hoarseness in group II and five cases in group I, but the difference between the two groups was not statistically significant (*p* > 0.05). There were 10 cases of cough in group II and 35 cases in group I, and the difference between the two groups was statistically significant (*p* < 0.05). See Figure 9.

### 3.6. Dislocation of the periportal end of the DLT in group II

When the patients in group II were moved from a horizontal to a lateral position, the periportal end of the DLT moved outward with an average distance of 1.5 ± 0.6 cm.

## 4. Discussion

In the present study, there were no differences between the two groups in general characteristics, and the DLT model was selected in such a way as to minimize the influence of the tube’s thickness (too thick or too thin) on the results.

Many previous studies have examined how to reduce the dislocation and misalignment of the bronchial cuffed end of the DLT during changes in a patient’s position. Karabiyik et al. [11] found that, in patients with total spinal rigidity resulting from compulsory spondylitis, position changes did not affect the position of a right-sided DLT due to limited head and neck mobility. In a study by Askins et al. [12], the application of a cervical collar similar to that used by Karabiyik et al. reduced neck flexion by 24%, extension by 30%, and flexion-compound extension by 49% after changing to a lateral position; it therefore also reduced the incidence of dislocation and misalignment of the bronchial cuffed end of the DLT. Most previous studies have focused only on preventing dislocation by limiting changes to the position of the head and neck, but these changes are unavoidable when the body position is changed. Moreover, it has previously been shown [4,13] that, during a change to a lateral position, the head and neck may rotate axially, resulting in dislocation of the bronchial cuffed end of the DLT even if the chin-synovial distance is kept constant. Benumof et al. [14] found that there might also be varying degrees of head and neck flexion or posterior tilt during the change from a horizontal to a lateral position, causing the DLT to move distally or proximally by a distance of up to 2.8 cm. If the DLT is firmly fixed at the periportal end according to the traditional method, the 2.8-cm shift is not possible at this end, so the shifted distance is transmitted to the bronchial cuffed end of the tube, forcing the cuff and the tracheal mucosa to undergo frictional dislocation, resulting in the dislocation and misalignment of the bronchial cuffed end of the tube.

In the present study, the rate of dislocation rate in group I was 44.6%, which was generally consistent with the data reported by Klein et al. [10]. However, the dislocation rate in group II decreased to 20.2%, which correlated with the fact that only the bronchial cuffed end of the DLT was inflated and fixed, not the periportal end. This might be caused by the fact that, although the head and neck flexes, tilts back, and rotates when the patient’s position is changed, the periportal end of the DLT can slide freely and smoothly in the oropharynx so that the dislocation occurs at that end of the tube, and the bronchial cuffed end of the tube is not displaced due to cuff inflation; thus, the dislocation and misalignment of the cuffed end of the tube is reduced.

There are two main factors that lead to tube dislocation and misalignment during the process of changing a patient’s position from horizontal to lateral: first, the forward flexion, backward tilt, and axial rotation of the head and neck; and second, the gravitational effect of the lungs and mediastinum in the lateral position, which causes a relative dislocation of the cuffed end of the DLT in relation to the bronchial opening. In the present study, it was observed that an average of 1.5 cm of outward dislocation occurred at the periportal end of the DLT in group II, which was generally consistent with the data reported by Benumof et al. [14]. The repeated movement of the tube in the trachea irritates the tracheal wall and the mucosa of the carina, producing hemodynamic fluctuations and aggravating injury to the tracheal carina, bronchial mucosa, and oropharynx [15]. In the present study, it was also observed that MAP and HR were significantly higher in group I after moving to a lateral position, but not in group II. The results of fiberoptic bronchoscopy after extubation in group II showed less injury to the tracheal carina and bronchial mucosa than in group I, and complications, such as sore throat and cough, were less severe in group II on the first postoperative day. This might be due to the fact that the periportal end of the DLT was not fixed in group II, allowing it to slide freely and smoothly in the oropharynx during position changes. This free movement might reduce the relative sliding friction between the cuffed end of the tube and the bronchial mucosa, which would not only reduce the dislocation and poor alignment of the bronchial cuffed end of the tube but also the irritation and injury of the tracheal mucosa and oropharynx and the effects on hemodynamics.

The present study had some limitations, mainly that there was no fixation at the peripheral end of the DLT, which increased the risk of accidental removal; such removal requires the anesthesiologist to change the patient’s position again. To address this limitation, the creation and implementation of a special dental pad and tube holder is planned for future work, which will allow the tube to slide freely and smoothly at the periportal end while also preventing its accidental removal.

## 5. Conclusion

The present study found that not fixing the periportal end of the DLT is less likely to cause dislocation and misalignment of the bronchial cuffed end when moving the patient from a horizontal to a lateral position. It is also less likely to influence hemodynamics and cause airway mucosal injury, which makes it more conducive to intraoperative anesthesia management and the reduction in incidence of postoperative complications.

## Data Availability

The datasets used and/or analysed during the current study available from the corresponding author on reasonable request.

## Clinical registration number

This study has been registered in the Chinese Clinical Trial Registry, Registration number: ChiCTR1900027200.
